# The Role of Multimodality Imaging in Monitoring Disease Activity and Therapeutic Response to Tocilizumab in Giant Cell Arteritis

**DOI:** 10.1155/2020/3203241

**Published:** 2020-09-27

**Authors:** Edoardo Conticini, Jurgen Sota, Paolo Falsetti, Caterina Baldi, Marco Bardelli, Francesca Bellisai, Gian Marco Tosi, Bruno Frediani, Luca Cantarini

**Affiliations:** ^1^Department of Medicine, Surgery and Neurosciences, Rheumatology Unit, University of Siena, Italy; ^2^Department of Medicine, Surgery and Neurosciences, Ophthalmology Unit, University of Siena, Italy

## Abstract

**Introduction:**

Giant cell arteritis (GCA) is a large vessel (LV) vasculitis, mainly affecting elder patients. Monitoring GCA activity during tocilizumab (TCZ) treatment is an unmet need, since low serum levels of C-reactive protein (CRP) during treatment may underestimate disease activity. To date, few data are available on the role of different imaging techniques in monitoring GCA activity and response to treatment. We report herein a cohort of GCA patients treated with TCZ and followed up with multimodal imaging. *Patients and Methods*. We collected clinical, laboratory, and imaging data of 11 GCA patients treated with TCZ 162 mg subcutaneously every week. Disease activity was assessed at baseline and within 12 months from the start of treatment using different imaging techniques such as color Doppler ultrasonography (CDUS), magnetic resonance imaging/angiography (MRI/MRA), computed tomography angiography (CTA), and/or positron emission tomography (PET).

**Results:**

Four patients were affected by cranial and 7 by LV-GCA. All patients were treated with oral glucocorticoids (GCs) (mean dose 55.68 mg ± 8.19 of prednisone or equivalent) in combination with TCZ. Treatment was preceded in 5 cases by 3 intravenous boluses of 1000 mg methylprednisolone. A significant decrease of the mean dose of oral GCs was observed between baseline and the last follow-up visit (4.65 ± 3.69 mg) (*p* = 0.003). TCZ treatment significantly decreased erythrocyte sedimentation rate (*p* < 0.01) and CRP levels (*p* < 0.01). At follow-up (mean 8.18 ± 3.63 months), all patients were in clinical and serological remission. Moreover, PET, CDUS, MRI/MRA, and CTA did not show any LVV finding.

**Conclusions:**

Our study highlights TCZ efficacy in inducing GCA remission and its steroid-sparing effect. We highlighted a reliability of imaging procedures in the evaluation of disease activity and treatment response. A close disease monitoring with imaging techniques should be taken into account in GCA patients during TCZ treatment.

## 1. Introduction

Large vessel vasculitis (LVV) is defined as an inflammatory involvement of large arteries, of which giant cell arteritis (GCA) and Takayasu arteritis represent the two main forms [1]. GCA is the most commonly encountered vasculitis among elder patients. The inflammatory process in GCA affects large- and medium-size vessels with a particular tropism toward cranial arteries derived from the carotid artery [1]. Associated aortitis can lead to aneurysm formation, rupture, or dissection, while luminal narrowing of the aorta's main branches can result in various ischemic complications.

GCA is classically associated with polymyalgia rheumatica (PMR), a common disorder among patients older than 65 which can precede the presentation of GCA even of several years [2].

Despite recent advances in imaging techniques, diagnostic process as well as disease monitoring represents unmet needs, especially in case of extracranial involvement and a nonspecific clinical spectrum [3].

Diagnosis of GCA classically relies on histological findings [4], but in the last years, imaging is becoming increasingly recognized as a reliable diagnostic tool. EULAR recommendations for the use of imaging in LVV stress the concept that imaging test should be performed as soon as possible, in order to refine diagnosis and prevent false-negative results after immunosuppressive therapy [5]. Nevertheless, no clear-cut guidelines address the use of imaging in monitoring disease activity and therapeutic response which is of paramount relevance in the biologic era. Given their unpredictable course and the increasing use of the newly introduced agents such as biologic disease-modifying antirheumatic drugs (bDMARDs), optimal follow-up of LVV is still far from being definite, since inflammatory markers may be unreliable in predicting disease flares. We herein report our monocentric experience on the use of several imaging techniques in monitoring therapeutic response to tocilizumab (TCZ).

## 2. Methods

This series comprises 11 adults (3 males, 8 females) affected by GCA and treated with TCZ 162 mg subcutaneously every week. Baseline clinical and radiological characteristics are given in Table 1. In 4 patients, affected by cranial GCA, diagnosis was performed according to 1990 American College of Rheumatology classification criteria [4], while in the other 7 by an experienced rheumatologist through clinical, serological, and radiological findings. Possible mimickers have been excluded beforehand. All patients undergoing biologic therapy were screened for active and latent infections by performing the following exams: complete blood chemistry, chest-X ray, QuantiFERON test, urine culture, and liver markers of HBV and HCV. Patient underwent color Doppler ultrasonography (CDUS) and/or magnetic resonance imaging/angiography (MRI/MRA) and/or computed tomography angiography (CTA) and/or 18F-fluorodeoxyglucose-positron emission tomography (FDG-PET) at the time of diagnosis, and the same procedure was repeated within 12 months from the start of treatment. PET findings were measured using standardized uptake value (SUV) and Meller scale.

The choice of the imaging was performed according to clinical reasons: PET was employed in all patients except the ones with an exclusive cranial disease, with no constitutional signs or symptoms suggesting LV involvement. On the other hand, CDUS was performed in all subjects with cranial symptoms and suspected involvement of supra-aortic trunks.

The aim of our study consisted in evaluating the response to TCZ therapy via several imaging techniques.

Wilcoxon signed-rank test was employed to evaluate glucocorticoid- (GC-) sparing effect.

## 3. Results

### 3.1. Patient 1

**Figure 1:**
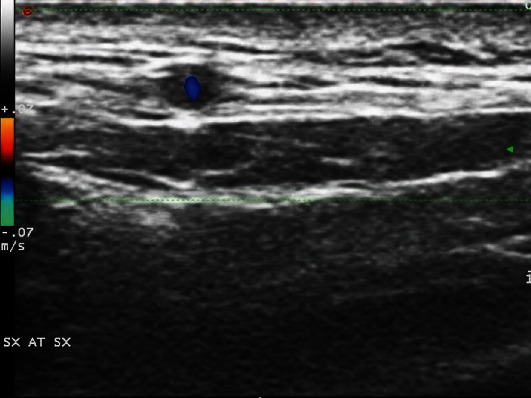
“Halo sign” of the left temporal artery.

A 65-year-old female patient, affected by dyslipidemia and coronary heart disease, who previously lost her right eyesight due to GCA, was referred to our Department due to left scalp tenderness, despite the treatment with low dosage of GCs. TA CDUS evidenced bilateral halo sign (Figure 1), with an intima-media thickness (IMT) of 0.7 cm, thus confirming a GCA relapse. After six months of treatment with TCZ, markers of inflammation were negative, as well as left TA CDUS, while halo sign persisted in the contralateral TA.

### 3.2. Patient 2

A 64-year-old female patient, with a previous diagnosis of PMR, reported scalp tenderness, fever, and malaise after steroid tapering. Due to inflammatory marker elevation, she underwent a metabolic evaluation with ^18^F-FDG PET, which showed a high FDG uptake (SUV max—5) of the axillary artery (AxA), subclavian artery (SA), and carotid artery (CA) and thoracic aorta. TA biopsy was negative. After three months of therapy with TCZ 162 mg and low doses of GC, PET was repeated, showing no uptake in the arterial tree. TA and supra-aortic trunk CDUS were negative too. Nevertheless, TCZ was stopped due to severe leukopenia.

### 3.3. Patient 3

**Figure 2:**
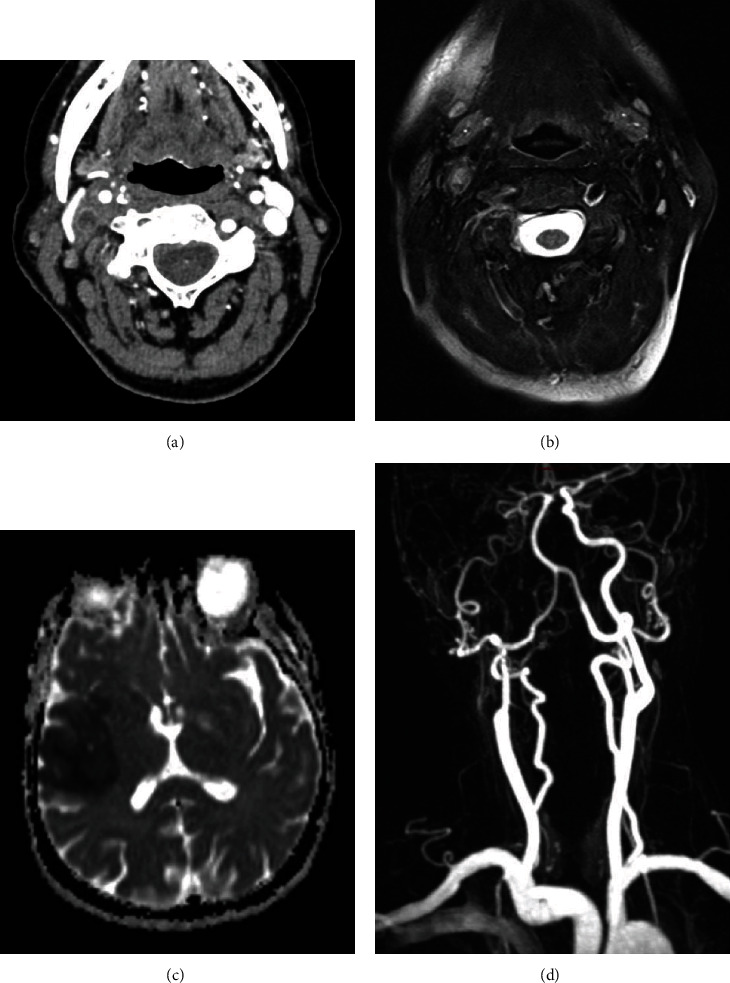
Axial postcontrast CT image (a) demonstrated the presence of vascular occlusion of the right internal carotid artery, immediately above the bifurcation, than confirmed by T2-weighted turbo spin echo (TSE) axial sequence that suggests also the presence of mural inflammation due to edema detection (b). Axial ADC subtraction sequence at the same time demonstrated the presence of a large frontotemporal ischemic lesion (c). Contrast-enhanced MR-angiography after therapy showed a reduction of the segmental occlusion however with persistent filiform contrasting of the right internal carotid artery (d).

A 70-year-old female, whose medical history was unremarkable, presented to the Emergency Department for persistent temporal headache, fatigue, fever, and sudden left arm paresis. MRA and cerebral angiography evidenced right internal CA severe occlusion and both common CA, left SA, and vertebral artery (VA) thickening. US showed concentric “halo” sign around the VA, SA, and CCA, with no flow in the territory of the right internal CA, while PET a SUV max of 2.74. Due to a scarce response to high dosage of GC, TCZ was added in the therapy. After six months, PET and CDUS were fully normalized, while MRI evidenced a partial recanalization of ICA and normal findings of the other vessels (Figure 2).

### 3.4. Patient 4

A 74-year-old female was evaluated for arthralgias, jaw claudication, scalp pain, and amaurosis fugax. Her inflammatory markers were elevated, and a SUV max of 3.9 was found in both SA. Due to GC inefficacy and adverse reaction to methotrexate (MTX), TCZ was prescribed, with a full resolution of PET findings after one year of therapy.

### 3.5. Patient 5

**Figure 3:**
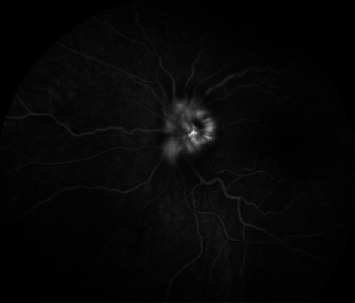
Late-phase angiogram showing leakage from the optic disk of the left eye.

A 78-year-old female, treated with low doses of GCs for PMR, was evaluated by our Ophthalmology Department for sudden visual loss, and arteritic anterior ischemic optic neuropathy (Figure 3) was diagnosed. Suspecting GCA, US was performed, with a halo sign of the TA. After three boluses of methylprednisolone (MPDN), TCZ was promptly started. CDUS and inflammatory markers were normalized, and patient considered in remission at three-month follow-up. However, TCZ was stopped due to a diagnosis of metastatic melanoma.

### 3.6. Patient 6, 7, and 8

**Figure 4:**
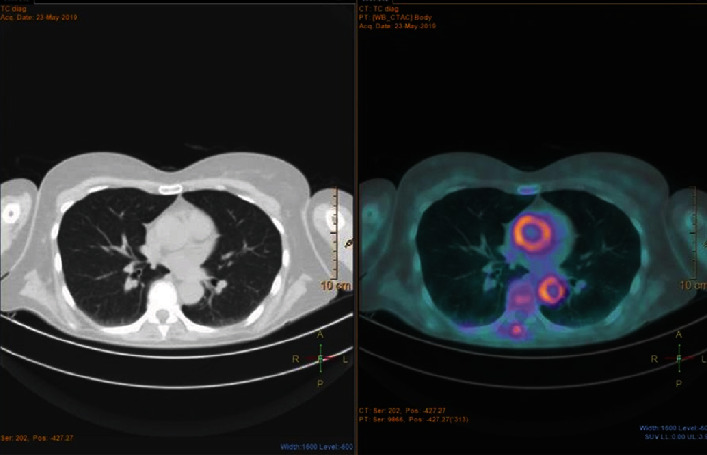
Diffuse ^18^fluorodeoxyglucose uptake in the territory of thoracic aorta.

**Figure 5:**
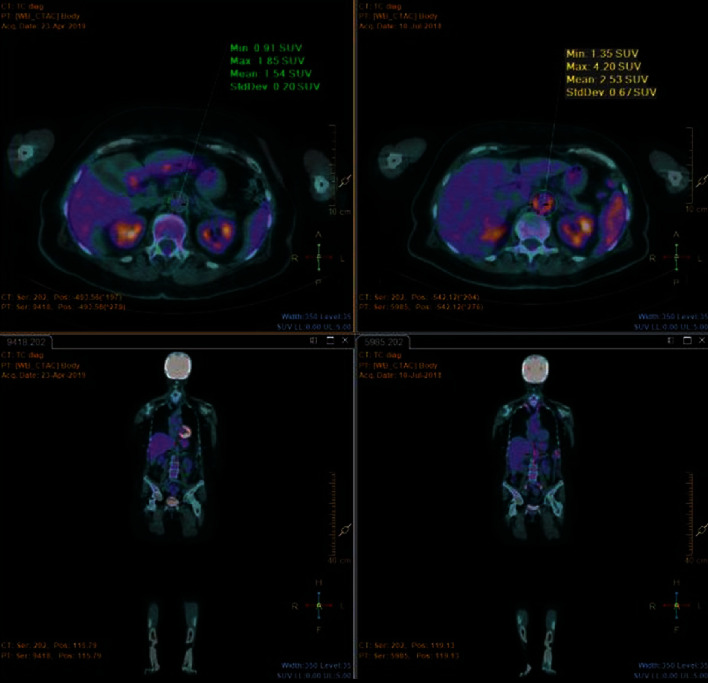
Abdominal aorta ^18^fluorodeoxyglucose uptake after (a) and before therapy (b).

Three patients, two females and one male, 56-, 77-, and 60-year-old, respectively, were referred to our Department for persistent fever, sweat, malaise, loss of weight, and anorexia, with inflammatory marker elevation. PET evidenced a high FDG uptake in the territory of the abdominal and thoracic aorta (Figure 4) and iliac arteries (IA), while CTA evidenced diffuse wall thickening. In two patients, TCZ was immediately prescribed in association with GC, while in the other one due to a relapse after intravenous cyclophosphamide (CYC). In all three subjects, all diagnostic procedures were repeated after one year of anti-IL-6 treatment: PET was normalized (Figure 5) while CTA evidenced no aneurysms or stenosis.

### 3.7. Patient 9

**Figure 6:**
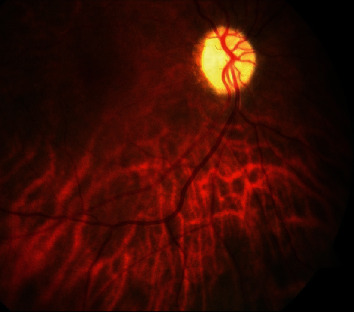
Retinography of the right eye showing optic atrophy as a consequence of arteritic optic neuropathy.

An 85-year-old diabetic patient, who reported sudden visual loss of the left eye a few months before, was evaluated due to a new episode affecting the contralateral eye (Figure 6), with inflammatory marker elevation. Temporal artery US evidenced halo sign, with an IMT of 0.7 cm. After three boluses of MPDN 1000 mg, TCZ was immediately prescribed, with poor visual acuity recovery. After 6 months of treatment, GCs were interrupted and CDUS evidenced an IMT of 0.3, while markers of inflammation were fully normalized.

### 3.8. Patient 10

A 72-year-old patient, affected by diabetes mellitus, hypertension, and monoclonal gammopathy, presented to the Emergency Department for diplopia and persistent headache. MRA evidenced right ICA stenosis and wall thickening, CDUS an IMT of 1.2 mm of ICA, and PET a slight FGD uptake in the supra-aortic trunks; thus, GCA was diagnosed. TCZ was subsequently prescribed, and after one year of therapy, all the abovementioned procedures were repeated: PET was negative, and ICA thickening, as seen in MRA and US, fully resolved.

### 3.9. Patient 11

**Figure 7:**
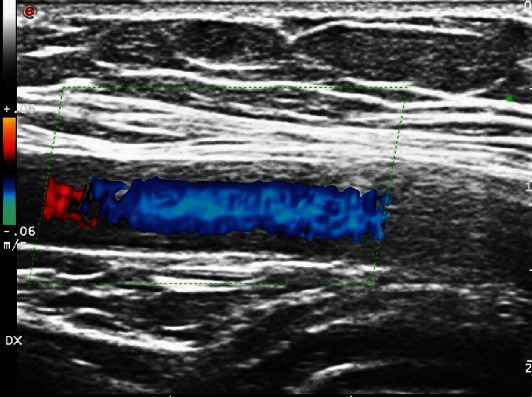
Intima-media thickening of the right axillary artery.

A 65-year-old patient was referred to our Department due to persistent dry cough and fever not responsive to antibiotics, with elevation of inflammatory markers. In the suspicion of LVV, he underwent US, which showed an IMT of 1.4 mm of both AxA (Figure 7), and PET, with FDG uptake (SUV max 4.38) in the territory of the aorta, IA, and supra-aortic trunks. TCZ and prednisone (PDN) 1 mg/kg were promptly started, leading to a complete remission within six months, with negative PET and CDUS.

We collected a total of 11 patients (8 females, 3 males), with a mean age of 70.0 (SD ± 8.44). Four of them suffered from cranial GCA and 7 from LV-GCA. In 2 of them, diagnosis was made using PET; 3 using US; 1 using both PET and US; 3 using PET and CTA; 1 using PET, MRI, and US; and 1 using PET, MRI, US, and cerebral angiography.

In 3 patients, whose presenting symptom was sudden blindness, only temporal arteries were involved, while in the other 8, imaging procedures evidenced an involvement of the aorta and/or supra-aortic trunks: they reported constitutional symptoms, such as fever, weight loss, fatigue, and night sweat, except one whose presenting symptom was a stroke leading to sudden hemiplegia. PMR was reported in 5 out of 11 subjects, and in 3 of them, PMR was diagnosed within the last 12 months.

At diagnosis, average erythrocyte sedimentation rate (ESR) and C-reactive protein (CRP) were 85 ± 23.71 mm/h and 6.76 *mg*/*dl* ± 4.08, respectively. Five patients were treated at baseline with three intravenous boluses of MPDN (1000 mg daily), followed by oral GCs in association with TCZ. The other 6 patients were treated with a dosage of oral GCs ranging from 50 to 75 mg of oral PDN or equivalent.

SUV max at baseline ranged from 3.06 to 6 (Meller scale III). Two patients underwent MRA, and they both reported wall thickening and enhancement of right internal CA, which was completely occluded in the patient presenting with stroke and hemiplegia, and right external CA. CTA evidenced wall thickening of the abdominal and thoracic aorta and iliac arteries in three subjects, all with positive PET. TA and supra-aortic trunk CDUS, performed in 6 subjects, was positive in all of them.

At the last follow-up, all patients were in clinical and serological remission, with a significant reduction of ESR and CRP of 12.09 ± 12.95 mm/h (*p* < 0.01) and 0.17 ± 0.14 mg/dl (*p* < 0.01), respectively.

A significant decrease of the mean GC dosage between the baseline (55.68 *mg* ± 8.19) and last follow-up visit (4.65 *mg* ± 3.69) (*p* = 0.003) was found. Moreover, two of them were in corticosteroid-independent remission.

PET was repeated in all patients who underwent this procedure at baseline and was normal in all cases at follow-up (SUV < 2.0). MRI findings were resolved in one patient, while in the other one, despite the full normalization of the other vessels, internal CA was only partially recanalized. CTA, repeated in all patients who underwent this procedure at baseline, was fully negative, and no aneurysms or stenosis were detected. US, repeated in all 6 patients with pathological IMT at baseline, was the range of normality in all of them. Imaging findings at baseline and at control are summarized in Table 2.

TCZ was discontinued in two patients due to the development of adverse events (1 case of severe neutropenia and 1 case of metastatic melanoma).

## 4. Discussion

Promising advances have been reached in the diagnosis and treatment of GCA in the last years. TA biopsy has been considered the gold standard for the diagnosis for many years, but more recently, in daily clinical practice, less invasive and easier to access procedures are being preferred. CDUS is an easy, safe, repeatable, and sensitive tool for the diagnosis of cranial GCA [6], and rheumatologists should routinely and rapidly perform it in case of clinical suspect. CDUS is particularly useful in the evaluation of AxA, SA, and CA too but has limited access for the aorta and abdominal vessels. In these conditions, MRI/MRA and CTA provide a higher diagnostic accuracy [7].

Finally, FDG-PET seems to have the highest sensitivity in detecting large vessel (LV) GCA [8]. Nevertheless, several shortcomings should be mentioned such as difficulties to visualize TA with this technique, due to their small diameter, their superficial location, and the vicinity of the glucose-consuming brain [5].

Although GCs remain the cornerstone of the treatment, more safe and powerful drugs may achieve a prompt and persistent remission and reduce the cumulative steroid dosage. Additionally, long-term liabilities of chronic especially in elder patients limit their usage rate.

TCZ, a humanized monoclonal antibody inhibiting both soluble and membrane-bound forms of IL-6 receptor [9], to date, is the only bDMARD approved for the treatment of GCA, supported by a growing body of robust evidences. Namely, a phase II study by Villiger et al. [10] followed by a phase III GIACTA study [11], found a significantly high relapse-free survival and sustained remission rate in the group treated with TCZ. In GIACTA trial, remission was defined as the absence of signs and symptoms attributable to GCA and normalization of ESR and CRP.

TCZ plays a key role in the blockage of the pathological pathways leading to the development of the disease, but, on the other hand, its mechanism of action makes monitoring of the disease using “classical” inflammatory markers quite doubtful. CRP is an acute-phase protein produced by the liver in response to inflammatory stimuli, particularly via IL-6; thus, low serum CRP levels during TCZ treatment may underestimate disease activity.

This unmet need has led to the research of new biomarkers unrelated to the IL-6 pathway [12–14], but their reliability needs to be verified in larger studies.

The abovementioned considerations, as well as the paucity of symptoms in case of LV involvement, may suggest reconsidering the concept itself of GCA “clinical and biochemical remission” mentioned by the current available recommendations [5]. Several studies have reported the cases of patients whose disease, although considered in stable remission, was fully active, despite a concomitant immunosuppressive therapy: evidence of active vasculitis has been found in an autopsy of a patient receiving TCZ and appearing in remission [15].

In our study, we enrolled 11 GCA patients treated with TCZ who underwent CDUS and/or PET and/or CTA and/or MRI/MRA at baseline and at follow-up evaluation within 12 months from the start of the treatment.

At follow-up, all patients were in clinical, serological, and radiological remission and two of them had discontinued oral PDN. GC dosage significantly decreased, thus highlighting a significant steroid-sparing effect of TCZ.

In our cohort, all patients were considered in remission and their clinical and serological findings, which were not surprising, were interestingly confirmed by the objective improvement and/or full resolution of the radiological data. The goal of a rapid remission was obtained in all patients within a few months after TCZ starting, with a rapid tapering of GCs and with no serious or unexpected adverse events.

In light of these findings, we advise to repeat also major diagnostic imaging procedures during follow-up since inflammatory markers may be unreliable in revealing LVV flares, especially during TCZ therapy. Moreover, long-term complications of LVV, such as aneurysms and stenosis, cannot be diagnosed without CTA and MRI, potentially leading to dreadful outcomes in therapy.

In particular, FDG-PET and MRA, alone and combined [16], could represent the best choice for monitoring LVV. FDG-PET provides the highest sensitivity for the assessment of mural inflammation, and their relatively low dose of radiation makes it suitable for repetitive scans. On the other hand, MRA, although time-consuming and expensive, is essential for the assessment of intracranial arteries and vessel wall, as well as in the monitoring of the common, and unpredictable, onset of long-term vascular complications (e.g. aneurysms and stenosis) [17], as mentioned in EULAR recommendations.

Nevertheless, we must remind that the role itself of PET during follow-up should be further evaluated and is still a matter of debate [18]; it is well-known that FDG decreases after an effective treatment, [19] but a persistent FGD uptake may be variously interpreted as a persistent disease activity as well as a sign of reparatory processes arising within vessel walls [20].

Uncertainty remains also in the interpretation of MRA findings during follow-up: in the only randomized control trial of GCA treated with TCZ and monitored with repeated MRA, signals normalized in only one-third of patients, despite being all considered in remission [21].

Finally, CDUS, easy to perform, not expensive nor time-consuming, may be considered a promising tool in the monitoring of GCA disease activity: nevertheless, uncertainty remains about its use in AxA, besides TA [22].

There is a number of limitations in our study: first, the low number of patients, so further confirmations from larger, multicentric studies are needed.

Secondly, we retrospectively collected our cases, leading to a considerable variety among the patients, as well as in the heterogeneity among diagnostic techniques performed at baseline and during follow-up.

Finally, the short term of our observational data cannot be considered sufficient to demonstrate a lower progression of aneurysms and stenosis in patients treated with TCZ.

Larger studies are urgently needed to clarify a shared protocol of monitoring of these patients and to assess the long-term efficacy of bDMARDs in providing a persistent remission and preventing further complications. In our opinion, according to our experience, a potential follow-up scheme may be performing CDUS at every follow-up visit or as needed, asking for more invasive, expensive, and time-consuming procedures, such as PET and CTA, every 12 months.

In conclusion, our study shows TCZ efficacy in reducing vascular inflammatory involvement as detected by CDUS and/or PET and/or MRA and/or CTA.

Imaging is becoming increasingly recognized as a reliable diagnostic tool, but we think that this reliability should be evaluated and potentially extended also in assessing disease activity and monitoring therapeutic response at different time points of follow-up. A close disease monitoring also with imaging techniques is warranted to optimize patient's management.

## Figures and Tables

**Table 1:** Patient clinical and serological features before and after tocilizumab treatment.

Pt	Gender	Age	ESR (mm/h) at baseline	ESR (mm/h) at follow-up	CRP (mg/dl) at baseline	CRP (mg/dl) at follow-up	Oral PDN dose at baseline (mg)	Oral PDN dose at follow-up (mg)	Follow-up (months)	AE leading to discontinuation
1	F	65	62	5	4.3	0.2	50	5	6	—
2	F	64	111	33	14.8	0.44	50	5	3	Leukopenia
3^∗^	F	70	62	6	4.35	0.08	50	5	6	—
4	F	74	80	2	6.9	0.1	50	5	12	—
5^∗^	F	78	50	2	5.08	0.06	62.5	12.5	3	Metastatic melanoma
6	F	56	103	5	14.1	0.04	50	2.5	12	—
7^∗^	F	77	89	2	6.73	0.08	62.5	0	12	—
8^∗^	M	60	53	31	1.24	0.21	62.5	1.25	12	—
9^∗^	F	85	120	4	4	0.01	50	0	6	—
10	M	77	103	35	8.9	0.44	50	5	12	—
11	M	65	102	8	4	0.3	75	10	6	—

AE: adverse events; CRP: C-reactive protein; ESR: erythrocyte sedimentation rate; PDN: prednisone; ^∗^patients who underwent three boluses of 1000 mg methylprednisolone before being treated with tocilizumab and oral PDN.

**Table 2:** Patient imaging findings before and after tocilizumab treatment.

Pt	Gender	Age	PET (Meller scale) at baseline	PET (Meller scale) at follow-up	MRA at baseline	MRA at follow-up	CTA at baseline	CTA at follow-up	CDUS IMT at baseline (mm)	CDUS IMT at follow-up (mm)
1	F	65	—	—	—	—	—	—	0.7 (TA)	0.4 (TA)
2	F	64	III	0	—	—	—	—		0.7
3	F	70	III	0	Right ICA occlusion; CCA, left SA, and VA thickening	ICA partially recovered; other arteries fully resolved	—	—	1.5 (CCA)	0.7 (CCA)
4	F	74	III	0	—	—	—	—	—	—
5	F	78	—	—	—	—	—	—	0.6 (TA)	0.2 (TA)
6	F	56	III	0	—	—	Aortic wall thickening	Negative	—	—
7	F	77	III	I	—	—	Aortic wall thickening	Negative	—	—
8	M	60	III	I	—	—	Aortic wall thickening	Negative	—	—
9	F	85	—	—	—	—	—	—	0.7 (TA)	0.3 (TA)
10	M	77	III	0	ICA enhancement	Negative	—	—	1.2 (CCA)	0.7 (CCA)
11	M	65	III	I	—	—	—	—	1.4 (AxA)	0.8 (AxA)

AxA: axillary artery; CCA: common carotid artery; CDUS: color Doppler ultrasonography; CTA: computed tomography angiography; ICA: internal carotid artery; IMT: intima-media thickness; MRA: magnetic resonance angiography; PET: positron emission tomography; SA: subclavian artery; SUV: standardized uptake value; TA: temporal artery; VA: vertebral artery.

## Data Availability

The datasets generated for this study are available on request to the corresponding author.

## References

[1] Jennette J. C., Falk R. J., Bacon P. A. (2013). 2012 revised International Chapel Hill Consensus Conference Nomenclature of Vasculitides. *Arthritis and Rheumatism*.

[2] Buttgereit F., Dejaco C., Matteson E. L., Dasgupta B. (2016). Polymyalgia rheumatica and giant cell arteritis: a systematic review. *JAMA*.

[3] Monti S., Floris A., Ponte C. (2018). The use of ultrasound to assess giant cell arteritis: review of the current evidence and practical guide for the rheumatologist. *Rheumatology (Oxford)*.

[4] Hunder G. G., Bloch D. A., Michel B. A. (1990). The American College of Rheumatology 1990 criteria for the classification of giant cell arteritis. *Arthritis and Rheumatism*.

[5] Dejaco C., Ramiro S., Duftner C. (2018). EULAR recommendations for the use of imaging in large vessel vasculitis in clinical practice. *Annals of the Rheumatic Diseases*.

[6] Luqmani R., Lee E., Singh S. (2016). The role of ultrasound compared to biopsy of temporal arteries in the diagnosis and treatment of giant cell arteritis (TABUL): a diagnostic accuracy and cost-effectiveness study. *Health Technology Assessment*.

[7] Guggenberger K. V., Bley T. A. (2018). Magnetic resonance imaging and magnetic resonance angiography in large-vessel vasculitides. *Clinical and Experimental Rheumatologp Rheumatol*.

[8] Nielsen B. D., Hansen I. T., Kramer S. (2019). Simple dichotomous assessment of cranial artery inflammation by conventional 18F-FDG PET/CT shows high accuracy for the diagnosis of giant cell arteritis: a case-control study. *European Journal of Nuclear Medicine and Molecular Imaging*.

[9] Okuda Y. (2008). Review of tocilizumab in the treatment of rheumatoid arthritis. *Biologics: targets & therapy*.

[10] Villiger P. M., Adler S., Kuchen S. (2016). Tocilizumab for induction and maintenance of remission in giant cell arteritis: a phase 2, randomised, double-blind, placebo-controlled trial. *Lancet*.

[11] Stone J. H., Tuckwell K., Dimonaco S. (2017). Trial of tocilizumab in giant-cell arteritis. *The New England Journal of Medicine*.

[12] van Sleen Y., Sandovici M., Abdulahad W. H. (2019). Markers of angiogenesis and macrophage products for predicting disease course and monitoring vascular inflammation in giant cell arteritis. *Rheumatology (Oxford)*.

[13] Prieto-González S., Terrades-García N., Corbera-Bellalta M. (2017). Serum osteopontin: a biomarker of disease activity and predictor of relapsing course in patients with giant cell arteritis. Potential clinical usefulness in tocilizumab-treated patients. *RMD open*.

[14] Gloor A. D., Yerly D., Adler S. (2018). Immuno-monitoring reveals an extended subclinical disease activity in tocilizumab-treated giant cell arteritis. *Rheumatology (Oxford)*.

[15] Unizony S., Arias-Urdaneta L., Miloslavsky E. (2012). Tocilizumab for the treatment of large-vessel vasculitis (giant cell arteritis, Takayasu arteritis) and polymyalgia rheumatica. *Arthritis care & research*.

[16] Einspieler I., Thürmel K., Pyka T. (2015). Imaging large vessel vasculitis with fully integrated PET/MRI: a pilot study. *European Journal of Nuclear Medicine and Molecular Imaging*.

[17] Kebed D. T., Bois J. P., Connolly H. M. (2018). Spectrum of aortic disease in the giant cell arteritis population. *The American Journal of Cardiology*.

[18] Salvarani C., Soriano A., Muratore F., Shoenfeld Y., Blockmans D. (2017). Is PET/CT essential in the diagnosis and follow-up of temporal arteritis?. *Autoimmunity Reviews*.

[19] Muratore F., Pipitone N., Salvarani C., Schmidt W. A. (2016). Imaging of vasculitis: state of the art. *Best Practice & Research. Clinical Rheumatology*.

[20] Blockmans D., De Ceuninck L., Vanderschueren S., Knockaert D., Mortelmans L., Bobbaers H. (2007). Repetitive 18-fluorodeoxyglucose positron emission tomography in isolated polymyalgia rheumatica: a prospective study in 35 patients. *Rheumatology (Oxford)*.

[21] Reichenbach S., Adler S., Bonel H. (2018). Magnetic resonance angiography in giant cell arteritis: results of a randomized controlled trial of tocilizumab in giant cell arteritis. *Rheumatology (Oxford)*.

[22] Ponte C., Serafim A. S., Monti S. (2020). Early variation of ultrasound halo sign with treatment and relation with clinical features in patients with giant cell arteritis. *Rheumatology (Oxford)*.

